# Supplementary material for: How did Ontario healthcare institutions implement and legitimize Covid-19 vaccine mandates? A qualitative multi‑method study protocol
Source: PLoS One. 2025 Oct 3;20(10):e0332589. doi: 10.1371/journal.pone.0332589 (PMC12494268; doi:10.1371/journal.pone.0332589)
Supplement: Attachment — Submitted filename: My response point by point.pdf [file pone.0332589.s001.pdf]

Point-by-Point Response to Reviewers

1. Reviewer comment: This is a really interesting, proposed project.

   Response: Thank you for taking the time to review my work and for your encouraging assessment.

2. Reviewer comment: The second research question may need to be reframed as stated now it does not open up the possibility that alternatives were promoted, or at the very least, tolerated in some cases. (i.e. what were the institutional rationales, and how were alternatives framed, dismissed, or suppressed?)

   Response: Thank you for your useful comment. If I understand you correctly you're asking me to reframe research question 2 to be more open-ended. I have revised question 2 to explicitly allow for documenting tolerance, accommodation, or promotion of alternatives to mandatory vaccination.

3. Reviewer comment: More details on sources for environmental scan would be useful, what specific documents will be included is there any type of inclusion/exclusion criteria?

   Response: Thank you for your observation. I have revised the section within methods corresponding to the access, selection, and inclusion exclusion criteria to clarify that the selected institutions are the access point and that the primary sources for the environmental scan will be official hospital policy documents describing COVID-19 vaccination mandates, as well as any explanatory or supporting documents, such as FAQs, HR bulletins, and public statements. I have also clarified that inclusion criteria are documents defining or explaining vaccine policy for staff and exclusion criteria are documents unrelated to vaccine mandates.

4. Reviewer comment: What is the selection for the criteria for 20-25 institutions.

   Response: Thank you for your observation. Upon revising the data section (as described earlier) I hope that my discussion of selection criteria is clearer now. In this section I clearly describe the sampling methodology from the initial list of the CBC database.

5. Reviewer comment: Where will information for phase 2 come from?

   Response: Thank you for your observation. I have clarified that Phase 2 will analyze the same documents gathered in Phase 1, applying a dissent-framing perspective, with no new data collection

6. Reviewer comment: How will you find instances of dissent from HCWs? especially if institutions are trying to suppress dissent, will this information be in publicly available documents? Would using other publicly available sources also support this phase of the research? Please clarify

   Response: Thank you for your observation. Under "research design/phase 2", I have clarified that the aim is not to identify hidden dissent but to analyze how institutions framed, justified, and responded to dissent in their public or semi-public documentation.
