# Supplementary material for: How did Ontario healthcare institutions implement and legitimize Covid-19 vaccine mandates? A qualitative multi‑method study protocol
Source: PLoS One. 2025 Oct 3;20(10):e0332589. doi: 10.1371/journal.pone.0332589 (PMC12494268; doi:10.1371/journal.pone.0332589)
Supplement: Attachment — Submitted filename: My response to reviewers.pdf [file pone.0332589.s002.pdf]

Reviewer 2 - optional suggestions and commentaries to the authors

Thanks for this interesting study protocol.

Thank you for your positive assessment of our study protocol. Please find below a point-by-point response to your comments. I appreciate your constructive suggestions and have revised the manuscript accordingly.

Here are some recommendations:

1. The study design does not constitute a typical mixed-methods study. Having "mixed-methods" in the title can be confusing for the reader and is best avoided.

Thank you for this helpful observation. I agree that our design is qualitative and that the term “mixed-methods” could be misleading. I have revised the title and all occurrences in the manuscript to describe the study as a qualitative multi-method protocol, consistent with methodological usage where “multi-method” denotes the combination of two or more qualitative methods (e.g., document analysis, environmental scan of institutional policies/websites, critical policy analysis) without the inclusion of quantitative components (see added reference: Roller, 2021). These changes better reflect the actual design and should avoid confusion for readers.

2. The background for the proposed work needs to include a discussion on healthcare vaccine mandates that predated and continued past the COVID-19 mandate. The existence of these mandates helped frame the justification for introducing the COVID-19 mandates and needs to recognized in this study.

Thank you for this thoughtful suggestion. I have added a short paragraph in the Introduction to contextualize the COVID-19 mandates in relation to prior influenza vaccine policies. While influenza mandates have occasionally been implemented in Canadian healthcare settings, they were typically less coercive and allowed for alternatives such as masking or redeployment. Most importantly, workers who declined influenza vaccination were not vilified, nor were those who complied valorized (see added references: BMJ 2013; Gruben, 2014; Edmond, 2019; Flood, 2021). In contrast, the COVID-19 mandates were accompanied by a dramatic shift in institutional tone and public framing — from heroism to threat — even though Ontario’s Directive 6 permitted multiple, far less intrusive pathways to compliance (e.g., an educational module and testing). This change highlights the need to examine not only policy content but also how it was enacted and justified at the institutional level.

3. Please clarify for the reader how much latitude individual hospitals had in interpreting the implementation process for the mandate issued by the Ontario Chief Medical Officer of Health. This is vital to understand why variability is to be expected in the different types of hospitals to be included in the study.

Thank you for this suggestion. I have clarified this point in the Introduction and throughout Phase 1. As revised, the manuscript now explains that Ontario’s Directive 6 mandated that all

covered organizations implement a COVID-19 vaccination policy but granted significant discretion to each institution in how to operationalize it. The Directive permitted multiple compliance options — including full vaccination, medical exemption, and participation in an educational session followed by regular antigen testing. While the Directive formally preserved institutional choice, most hospitals implemented only the most restrictive version. Our study explicitly investigates this pattern of selective implementation, its justification, and its implications. I believe the revised manuscript now clearly addresses your concern.

4. Consider updating the dates on the timeline table.

Thank you for this suggestion. The timeline has been updated to reflect the current stage of the project and align with the revised protocol. The changes are administrative and clarify the sequencing of activities between Phase 1 and Phase 2. No major design or methodological changes have been made. As before, our plan is to begin manuscript preparation and submission upon completion of each phase, rather than wait for the full project to conclude. This reflects our typical workflow, which prioritizes timely dissemination while maintaining continuity across the research program.

5. In the limitations and elsewhere, you mentioned documenting and explaining contestation of the mandates. Re, "document and explain how they were implemented, justified and contested". I remain uncertain as to where documentation of contestations will come from for this study.

Thank you for this important question. As clarified in the revised manuscript (see Introduction and Phase 1 description), we will document contestation through a diverse range of publicly available sources. These include: (1) legal challenges filed by employees or unions (e.g., arbitration rulings, grievances, court filings) retrieved via the Canadian Legal Information Institute (CanLII); (2) media coverage describing institutional conflicts, staff terminations, or episodes of internal dissent; and (3) hospital-generated materials (e.g., HR bulletins, FAQs, press releases) that may indirectly acknowledge contestation — for instance, by outlining consequences for noncompliance or providing justifications for disciplinary measures. While these sources do not capture all forms of dissent, they offer important insight into how hospitals publicly framed and managed noncompliance. These materials will form the basis for the interpretive phase of our study, which focuses on institutional discourse and the representation (or silencing) of contestation.
